# Modest deviations from optimal adherence to antiretroviral therapy promote residual HIV-1 replication in the absence of virological rebound in plasma

**DOI:** 10.1186/1742-4690-8-S2-O35

**Published:** 2011-10-03

**Authors:** Alexander O Pasternak, Marijun de Bruin, Suzanne Jurriaans, Margreet Bakker, Ben Berkhout, Jan M Prins, Vladimir V Lukashov

**Affiliations:** 1 Academic Medical Center of the University of Amsterdam, Medical Microbiology, Amsterdam, Netherlands; 2 University of Wageningen, Communication Science, Wageningen, Netherlands; 3 Academic Medical Center of the University of Amsterdam, Internal Medicine, Amsterdam, Netherlands

## Background

Modern antiretroviral therapy (ART) is assumed to allow a certain degree of nonadherence while still maintaining complete suppression of viral replication ("forgiveness"), as virological suppression, measured by the commercial plasma viral load assays, is common at adherence levels >55-70% [1]. Yet, it is unknown whether HIV-1 replication is completely suppressed at these levels of adherence. [2]Here we investigated whether modest non-adherence to ART influences levels of HIV-1 RNA and DNA in peripheral blood mononuclear cells (PBMC).

## Materials and methods

Levels of HIV-1 unspliced RNA (usRNA) and viral DNA were quantified by seminested real-time PCR [2-4] in PBMC of 40 HIV-infected patients who had been on successful ART for a median of 3.8 years before the start of the study and with good immune reconstitution (median baseline CD4^+^ count, 620 cells/mm^3^). For every patient, three longitudinal samples, taken with 3-4 month intervals, were analyzed. One-week mean adherence to ART (percentage of prescribed doses taken) prior to the sampling moments was measured electronically.

## Results

Adherence never fell below7O% in any patient, and concurrent plasma viral loads of 109/120 (91%) PBMC samples were undetectable (<50 cop/ ml); for 10/11 remaining samples they were <100 cop/ml. Longitudinally, 23 patients were constantly 100% adherent, eight demonstrated improving adherence in time, and nine ("poor adherers") showed decreasing, variable, or constantly <100% adherence. Notwithstanding the lack of virological rebound in any of the patients, poor adherence, but not optimal or improving adherence, caused a significant longitudinal increase in usRNA levels (*P*=0.006). Remarkably, the change in adherence patterns from optimal through improving to poor was paralleled by a gradual increase in the corresponding viral RNA trends. Significant differences between the poor adherers and the remaining patients were observed in time-weighted changes from baseline (*P*=0.0006) and regression slopes (*P*=0.009) of usRNA, but not of viral DNA. These effects were independent of the therapy regimen or the time of virological suppression. Same effects were observed in a subset of patients whose plasma viremia was constantly undetectable (n=30).

## Conclusions

As ART only blocks the infection of new cells, but not viral RNA transcription in infected cells, the observed effect of decreased ART pressure (resulting from decreased adherence) on HIV RNA levels in PBMC strongly suggests new replication cycles despite ART, and not simply enhanced HIV-1 transcription in cells infected prior to therapy initiation. Our results represent the first evidence indicating that constant optimal adherence to modern ART may be necessary to stop all HIV replication.
